# Acceleration of ion phase-space holes due to interactions with ion solitons in a wave-guided plasma

**DOI:** 10.1038/s41598-024-72316-z

**Published:** 2024-09-12

**Authors:** Allen Lobo, Vinod Kumar Sayal

**Affiliations:** Department of Physics, Sikkim Manipal Institute of Technology, Sikkim Manipal University, Sikkim, 737136 India

**Keywords:** Hole-soliton interaction, Ion hole acceleration, Ion phase-space hole, Plasma physics, Magnetically confined plasmas

## Abstract

Ion phase-space holes are solitary kinetic structures found in the ion phase-space of collision-less plasmas, and are nonlinear solutions to the Vlasov-Poisson equations, identified as Bernstein-Greene-Kruskal (BGK) modes. In this study, interactions between an ion phase-space hole and a travelling ion KdV soliton is presented. This interaction, which is simulated in a fully ionised highly magnetised plasma within a cylindrical wave-guide, exhibits acceleration and deacceleration of the ion hole, depending on its mode of collision with the travelling ion soliton. We present these interactions and discuss the mechanism of this interaction between the two solitary waves.

The domain of research in the fields of plasma physics is ubiquitously populated by the study of waves which are inherently nonlinear in their propagation and interaction mechanisms and exhibit a widespread variety in terms of their nature, formation techniques and their interactions with the plasma medium. Such interactions influence their stability, propagating speed, waveform and other structural characteristics, which often exhibit parametric dependence on the properties of the plasma. Electrostatic solitary waves are a typical class of such nonlinear waves which are often observed in both astrophysical and laboratory plasmas, travelling in the medium as bipolar spatial electrostatic fields. Korteweg-De Vries (KdV) solitons and phase-space holes are two well-known plasma waves which exhibit this solitary nature. While the former is a nonlinear wave solution to the fluid-like nature of the plasma system, the latter is a purely kinetic phenomena and is governed by the collision-less kinetic theory of plasma. Ion solitons are KdV solitons in one-dimensional ion plasma fluid and are a common occurrence in space plasmas, including Earth’s aurora and magnetosphere^1–4^. KdV solitons are known to conserve their waveforms during their propagation in a fluid, however, the kinetic approach to the problem presents their interaction with resonant particles in the medium and a nonlinear damping of their amplitude^5,6^. Various investigations using the kinetic approach have been performed which discuss their interactions with resonant particles in various environments, including cylindrical wave-guide loaded plasmas, which collectively portray a better picture of the soliton wave-dynamics than the classical fluid approach^7–11^. Phase-space holes, on the other hand, are nonlinear Bernstein-Greene-Kruskal (BGK) modes which appear as well-formed, stable, cusp-like depressions in the phase-space of particles in collision-less plasmas. They are nonlinear solutions to the Vlasov-Poisson system and are principally found as Debye-scaled bipolar electrostatic solitary waves in Earth’s outer atmosphere^12–15^. These are also well-known to occur as broadband electrostatic noise in magnetosphere observations^14,16^. Ion holes are phase-space holes in the plasma ion phase-space, and occur as negative monopolar potential solitary waves travelling with ion thermal speeds and exhibiting remarkable stability, and are well-known to form through ion streaming instabilities^17–20^.

In a well-known experimental set-up^21,22^, it was shown that when a cylindrical wave-guide, loaded with fully ionized plasma is excited by a step-potential, a solitary electron phase-space hole (a phase-space hole in the electron phase-space) is formed along-with a negative KdV soliton. Studies depicting the spatial characteristics and interaction mechanisms of the phase-space hole with another hole and with the travelling KdV soliton have been performed on the bases this experiment^14,17,23^, which was also reproduced in numerical simulations^21,22,24^. It was found that an electron hole and an electron KdV soliton pass through each other during their collision, without affecting each other’s propagation characteristics or behaviour^22,23^. Phase-space holes, which are often described as a charged cloud in the plasma^25^, also exhibit soliton-like phase-shifting behaviour in the ultra-fast (mach numbers $M>10$) regime during mutual collisions, as shown by Jenab et al.^26^. This indicates that phase-space holes can, at-least in a weak sense, be considered to have soliton-like nature, and motivates one to explore their interactions with travelling KdV wave solitons.

Inspired from this, we present in this paper a novel interaction between an ion hole and an ion KdV soliton, in a magnetised one-dimensional confined plasma. Using a technique similar to the Q-machine simulation of electron hole formation^21,24^, the formation of an ion phase-space hole in a cylindrical wave-guide loaded, highly magnetised plasma is presented. Upon employing a suitable excitation technique, we observe the formation of a slow, negative solitary wave and a fast, positive wave propagating along the axial direction. We identify the former as an ion phase-space hole and the latter as an ion KdV soliton. We then modify our excitation techniques appropriately and produce interactions between the ion hole and the ion soliton and present the observed results. We find, from this simulation study, some interesting behaviours of the ion hole during its interactions with ion solitons of nearly equal potential amplitudes – ion holes tend to accelerate in the presence of ion solitons. This behaviour of ion holes is, however, lost when the soliton amplitudes are considerably small, when compared to the absolute hole amplitudes. This study presents an interesting form of interaction mechanism between holes and solitons of nearly equal amplitudes. It also presents an interesting technique of hole accelerations using solitary potential waves. Such interactions may also be observed during the outer-atmospheric observations of ion solitons propagation and their interactions with ion holes which exists in such regions.

## Methods

It has been shown in previous works^21,23,26^ that due to the large differences between amplitudes and speeds of electron KdV solitons and electron phase-space holes, no substantial interactions between the two solitary waves could be reportedly observed. Ion phase-space holes travel with speeds near to the ion thermal speeds, where-as the KdV solitons known to travel with phase-speeds comparable to the ion acoustic waves. However, as shown by Ghosh and Das^8^, the phase-speeds of the ion acoustic solitons further reduces by a factor $\simeq 0.72$ during their propagation through a cylindrical wave-guide, as compared to an unbounded plasma. Works of Karpman et al.^5,6^, Sayal and Sharma^9^ and Jenab et al.^11^ also exhibit the interaction of ion solitons with resonant particles during their propagation in the cylindrical bounded plasma. These works therefore point towards the possibility of formation of slow moving ion solitons which can interact with a propagating ion hole in the cylindrical plasma, thus serving as the chief motivation for conducting this study in the cylindrical-confinement set-up.

### Experimental set-up

We consider a cylindrical wave-guide loaded with fully ionised plasma. Both singly charged ions and the electrons are initially in thermalised states. A step-potential perturbation is inserted along the plasma column length which increases and then decreases in a sinusoidal fashion in time. The excitation amplitude is chosen in terms of the ion thermal potential, and is sufficiently large such that an ion phase-space hole and an ion KdV soliton is produced. The spatial electrostatic field and potential of the plasma is plotted and correspondingly the ion phase-space is observed. Interactions between the hole and soliton are produced by introducing multiple excitation at different locations along the plasma column.

### Governing equations and numerical forms

This experimental arrangement, which has been simulated in this paper, consists of a fully ionised plasma in which electrons follow a thermal distribution,

1 $$\begin{aligned} n_e = \exp (e\phi (x)/K_{B}T_{e}). \end{aligned}$$

Here $n_e$, $-e$, $\phi (x)$, $K_B$ and $T_e$ are electron spatial density, electron charge, spatial electrostatic potential, Boltzmann’s Constant and electron temperature, respectively. A plasma column of long axial length ($\simeq 500\lambda _{Di}$ to $1000\lambda _{Di}$) and radius $a_0$ ($\simeq 5\lambda _{Di}$) is used, comprising singly ionised positive species. Here, $\lambda _{Di}$ represents the ion Debye length. Owing to the radial confinement, the Poisson equation includes an additional radial term which describes the contribution of the lowest radial eigenmode,

2 $$\begin{aligned} \frac{\partial ^2\phi }{\partial x^2} - k_{\perp }^2\phi (x) = -\frac{\rho (x)}{\epsilon _0},\quad \text {where}\quad k_\perp = \frac{2.404}{a_0}. \end{aligned}$$

Here $\rho (x)$ is the spatial charge density and $\epsilon _0$ is the spatial electrostatic permittivity. The $k_\perp$ term is the contribution of the lowest eigenmode of the radial expansion of the Laplacian in the cylindrical form, and has been commonly used for numerical as well as analytical studies by many authors^21–24,27^. The ions initially follow a Maxwellian distribution,

3 $$\begin{aligned} f_i(x,v_x) = \sqrt{\frac{m_i}{2\pi K_BT_i}}\exp \left[ -\frac{m_iv_x^2}{2K_BT_i}\right] . \end{aligned}$$

The electron-ion temperature ratio $(T_e/T_i)$ in this work is taken to be 100.0. Axial boundaries are taken to be reflective, however, wave-absorbing plates are applied at the boundaries during the simulation during long-term evolution of the system. This is done in order to prevent formation of large amplitude nonlinear structures from the evolution of the formed rarefaction waves, which occurs during long-time evolution of the simulation.

### Excitation technique

In order to form solitary structures in the phase-space, a spatio-temporal perturbation is introduced into the system in the form of a potential field $\Phi (x,t)$:

4 $$\begin{aligned} \Phi (x,t)&= A\cdot \eta (x)\cdot \sigma (t),\quad \text {where,} \end{aligned}$$

5 $$\begin{aligned} \eta (x)&={\left\{ \begin{array}{ll} 0 & x\le x_0 \\ \frac{1}{2}\left[ 1-\cos \left( \frac{x-x_0}{\delta }\right) \right] & x_0\le x\le x_0+\delta \\ 1& x\ge x_0+\delta . \end{array}\right. } \end{aligned}$$

6 $$\begin{aligned} \sigma (t)&= \frac{1}{2}\left[ 1-\cos (2\pi t/t_0) \right] , \quad (t<t_0) \end{aligned}$$

**Figure 1:**
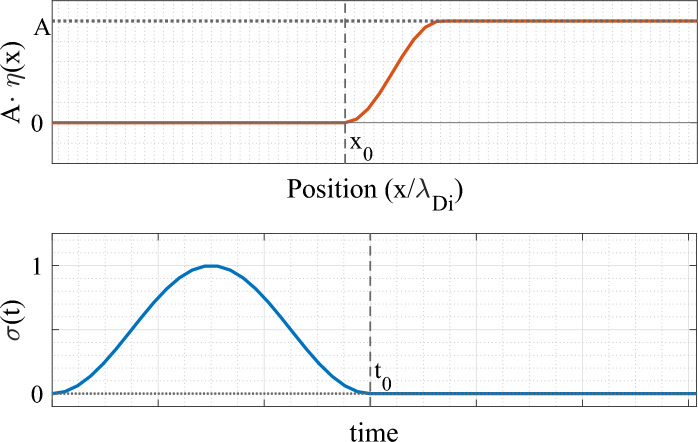
Graphical representation of the excitation perturbation functions used to excite ion holes and ion solitons in the plasma, similar to the excitation techniques developed by Turikov^24^. (Above)–spatial function $\eta (x)$, with $x_0$ as the position of the excitation and *A* the amplitude of excitation. (Below) time-profile $\sigma (t)$ of the perturbation, which starts at $t=0$, increases and gradually stops at $t=t_0$.

Here the perturbation amplitude *A*, width $\delta$, perturbation position $x_0$ and perturbation time $t_0$ are predefined parameters. The excitation functions $\eta (x)$ and $\sigma (t)$ are shown in Fig. 1.

The amplitude of the perturbation is chosen to be at-least equal to the ion thermal potential $(K_BT_ie^{-1})$. We see that for $A<<K_BT_ie^{-1}$, trapping does not occur and an ion hole is not formed. Perturbation width $\delta$ is chosen to be equal to $2\lambda _{Di}$ throughout the simulation study. This technique of perturbation is similar to the excitation technique of a Q-machine plasma developed by Turikov^24^. The kinetic simulation of the set-up is performed using numerical integration of the Vlasov-Poisson equation system–

7 $$\begin{aligned} \begin{aligned} \frac{\partial f}{\partial t} + v_x\frac{\partial f}{\partial x}- \frac{e}{m}\frac{\partial \phi }{\partial x}\frac{\partial f}{\partial v_x}&=0,\\ \frac{\epsilon _0}{e} \left( \frac{\partial ^2\phi (x)}{\partial x^2} - k_\perp ^2 \phi (x)\right)&=n_e - n_0\int _{-\infty }^{\infty }f_i(x,v_x)dv_x.\\ \end{aligned} \end{aligned}$$

For the same, the finite splitting scheme developed by Cheng and Knorr^28^ is employed, along-with a cubic spline interpolation technique for phase-space density shifting at the half-time intervals. In the numerical equations, we normalise the quantities with appropriate plasma parameters. Specifically, position is normalised by the ion Debye length $\lambda _{Di} = \sqrt{\frac{\epsilon _0K_BT_i}{n_0e^2}}$, time with the inverse of ion plasma frequency $\omega _{pi} = \sqrt{\frac{n_0 e^2}{\epsilon _0 m_i}}$, and velocity with the ion thermal velocity $v_{Ti} = \sqrt{\frac{2K_BT_i}{m_i}}$. Other physical quantities are normalised accordingly.

### Simulation code

For the simulation of the experiment, we have developed a grid-based kinetic Vlasov simulation code. The same can be collected from the corresponding author (A.L.) upon reasonable request. The code initially simulates a Maxwellian distribution ion phase-space, and evolves it by introducing the potential perturbation.

## Results

The numerical simulation of the plasma set-up is performed and depending on the perturbation amplitude, formation of ion phase-space holes and ion solitons are observed. Ion holes are formed only for sufficiently large amplitude perturbations, and remain nearly stable throughout the simulation. On the other hand, the ion solitons exhibit damping of their amplitudes and a corresponding growth of their spatial widths. We then modify our perturbation techniques and produce head-on and overtaking collisions between ion solitons and ion holes. Here we initially present the observations of a single hole and soliton formation and propagation, followed by the interaction simulations.

### Formation of an ion KdV soliton and an ion phase-space hole

**Figure 2:**
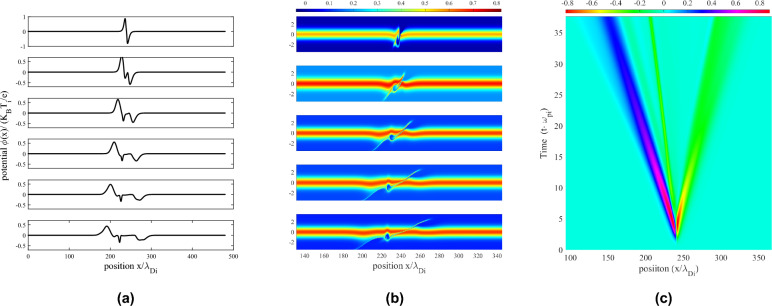
Formation and propagation of an ion phase-space hole (negative pulse moving to the left) and an ion KdV soliton (positive pulse moving to the left). (**a**) Potential fields at time steps of $3.4288 \omega _{pi}^{-1}$ (starting from top, time $=3.4288 \omega _{pi}^{-1}$) and (**b**) corresponding phase-space density portraits. (**c**) Spatio-temporal portrait of wave potentials exhibiting wave propagation. Ion soliton (pink-blue wave) and ion hole (thin, green wave propagating to left) can be seen. Stability of ion hole potential amplitude is shown from the color gradient, whereas the ion soliton exhibits a decay in its amplitude.

**Figure 3:**
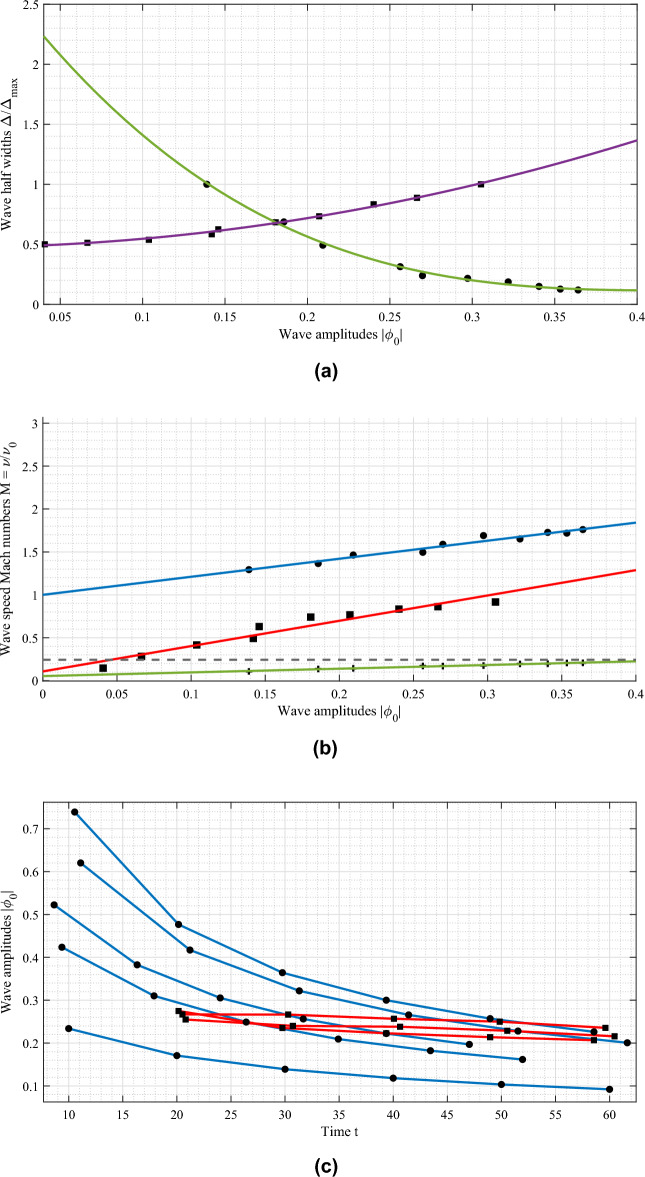
Ion KdV soliton (black circle) and ion hole (black square) characteristics. (**a**) Amplitude vs. half-width, (**b**) wave speeds of ion solitons and ion holes. Comparison of speed-amplitude ratio for simulated waves and the linear approximation from analytical study^8,29–31^. (**c**) Ion soliton and hole wave amplitudes vs. time during propagation. In the graphs, position normalised by ion Debye length $\lambda _{Di}$, potential by ion thermal potential $K_BT_i/e$, time by inverse ion plasma frequency $\omega _{pi}$ and speed by ion thermal speed $V_{Ti}$.

Figures 2a–c show the time-evolution of the plasma potential and the ion phase-space. The formation of a solitary ion phase-space hole is shown as the slow, negative solitary wave travelling along position space, and the associated phase-space density can be seen with the cusp-like depression at the site of the phase-space hole. Similarly, the positive, fast ion soliton is shown, which produces a hump-like local shift of the phase-space density function. It can be seen that during the propagation, the amplitude of the phase-space hole (shown in Fig. 2a,c as the thin, negative (green) wave travelling along $(-x)$ axis) remains almost stable, where-as the ion soliton (blue, laterally expanding wave in Fig. 2c) experiences damping of its amplitude. This is in agreement with the studies conducted by Karpman et al.^5,6^. The decay in amplitude and increase in over-all width of the soliton does not affect its speed, which remains a constant. Figure 3a–c show the width-amplitude-speed relationships of the propagating ion soliton and ion hole generated using different perturbation amplitudes. For the same, the wave half-width is estimated by the distance at which the wave potential reduces to half of its peak value.

From the graph 3a, it can be seen that the hole width (purple curve, square points) increases with its amplitude, which is typical to hole solutions^15,32,33^, where-as the ion soliton width (green curve with circular points) is inversely dependant on its amplitude, which is a signature characteristic of KdV solitary wave solutions. Figure 3b portraits the comparison between ion soliton speeds (blue line, black circular points), the speed-amplitude ratio (solid green curve) its comparison to the linear analytical approximation^29–31^ as shown by Ghosh and Das^8^ (black, dashed line) and ion phase-space hole speeds (red line with square points). The wave speeds ($\nu$) are presented in units of the perturbation speed $(\nu _0)$. For an ion hole formation, the perturbation amplitude must be at-least equal to the ion thermal potential.

**Figure 4:**
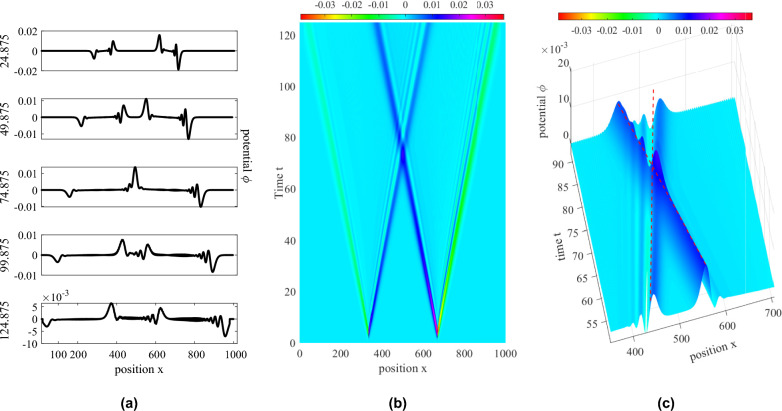
Head-on collision of small-amplitude ion KdV solitons. (**a**) Spatial potential profiles at time steps (mentioned on the left, normalised with inverse ion plasma frequency). (**b**) Spatio-temporal portrait of wave potentials (blue for solitons and green for the inserted perturbations) and (**c**) Associated phase-shift of the solitons during their collision.

There exists a linear relationship between the speeds and amplitudes of the ion hole and ion soliton formed from the presented perturbation. Specifically, there exists a relative amplitude difference $\Delta |\Psi _0|$ of value approximately equal to 0.1 between the ion hole and the ion soliton for small amplitude perturbations. This difference gradually decreases by increasing the perturbation amplitude, such that large amplitude holes are formed, which are closer to the amplitude of the ion solitons. Similar differences in the soliton and hole speeds also exist, which can be seen to decrease with increase in the amplitude of the perturbation. This suggests that upon using two perturbation pulses of sufficiently large but close amplitudes, interactions between holes and solitons of nearly equal amplitudes can be observed. Figure 3c shows the damping of soliton amplitudes (blue curves) and the relative stability of the hole amplitudes^14,25^ (red curves). The temporal damping of the ion soliton amplitude can be seen to be similar to the analytical findings^5,6^, and reduces in long-time scales. We then introduce collisions between two travelling solitons, as shown in Fig. 4a–c. As is expected, the solitons pass through each other without affecting each others’ propagation characteristics, and exhibiting the characteristic phase-shift behaviour.

Results from Fig. 3a–c show that with sufficiently strong and nearly equal perturbations, an ion-soliton and an ion hole of nearly equal wave characteristics can be produced in the wave-guide loaded plasma. Interactions between ion holes and ion KdV solitons can be performed by simulating head-on and overtaking collisions between the two solitary waveforms and varying their amplitudes and speeds. We present these interactions and their results in the next section. We observe that while there is no specific range of ion hole amplitudes for which these interactions occur, the interaction time is dependent on the soliton and hole potential amplitudes. We discuss this relationship later in the next sections.

### Interaction between ion solitons and ion holes

In order to produce mutual interactions between the formed ion solitons and ion phase-space holes, we modify our perturbation techniques such that pulses are presented simultaneously near opposite ends (for head-on collisions) and at the same point in the plasma column after some time interval (for overtaking collisions). The amplitudes of the solitary waves are regulated by the perturbation amplitudes. We observe and report an interesting difference in the interactions of both head-on collisions and overtaking collisions between the ion holes and ion KdV solitons, which we discuss later in the next section.

**Figure 5:**
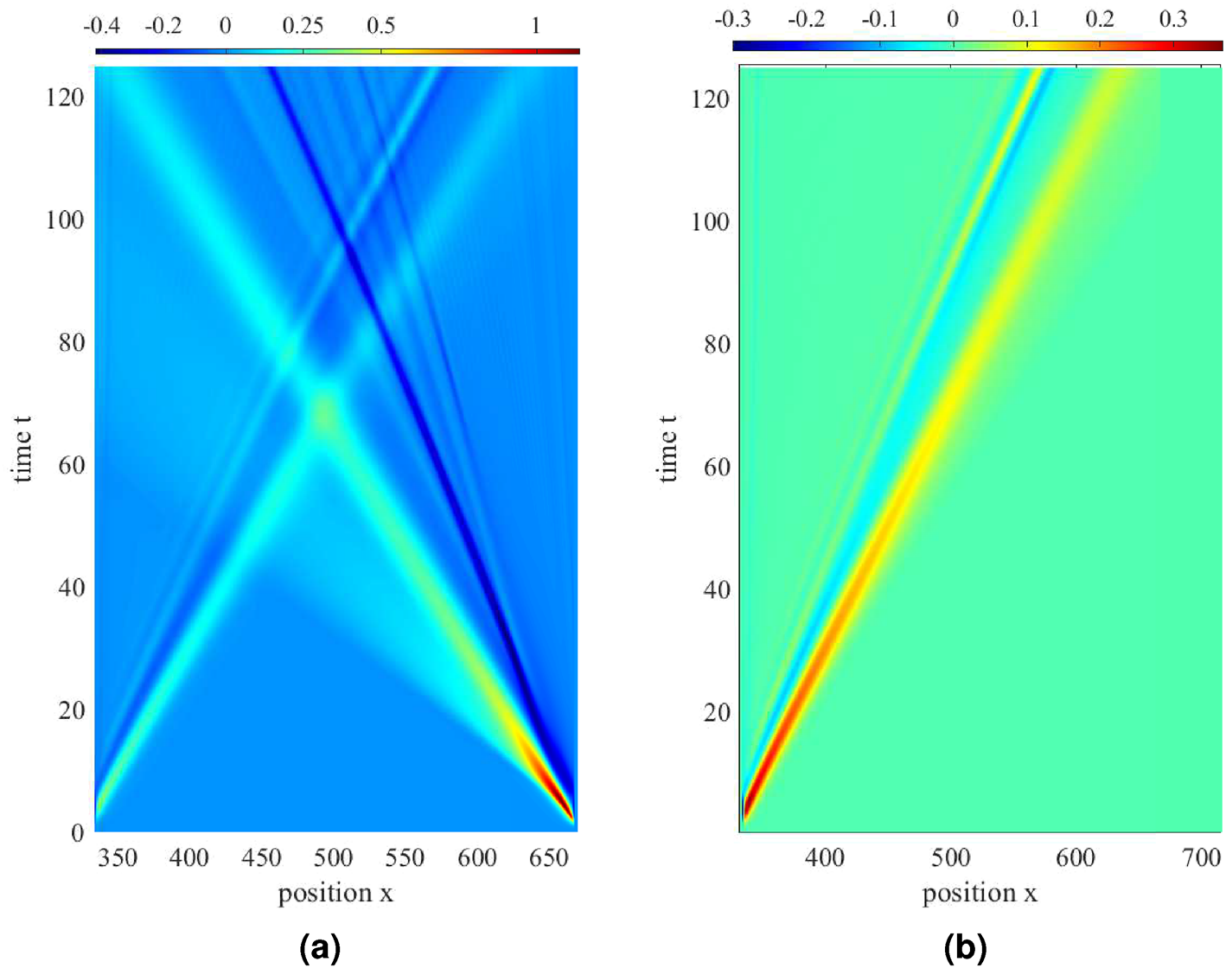
Head-on collision between an ion phase-space hole (negative potential wave, dark blue) and a small amplitude ion KdV soliton (positive potential wave, bright blue-yellow). (**a**) Propagation of the ion soliton in the presence of an ion hole and (**b**) absence of ion hole. It can be seen that the two solitary waves simply pass through each other, without affecting each other’s propagation dynamics.

We start our analyses by first demonstrating the interaction between a small amplitude ion soliton (thick, bright) and a large (negative) amplitude ion phase-space hole (thin, dark-blue streak), as shown in Fig 5a,b. It can be seen that the small amplitude, large-width soliton, which further reduces in its amplitude due to the present collision-less damping, does not perceptibly impact the propagation of the ion hole, which simply passes through the soliton, conserving its amplitude and speed (see Fig. 5a,b). It can also be seen that the ion soliton also remains unaffected by the ion hole.

**Figure 6:**
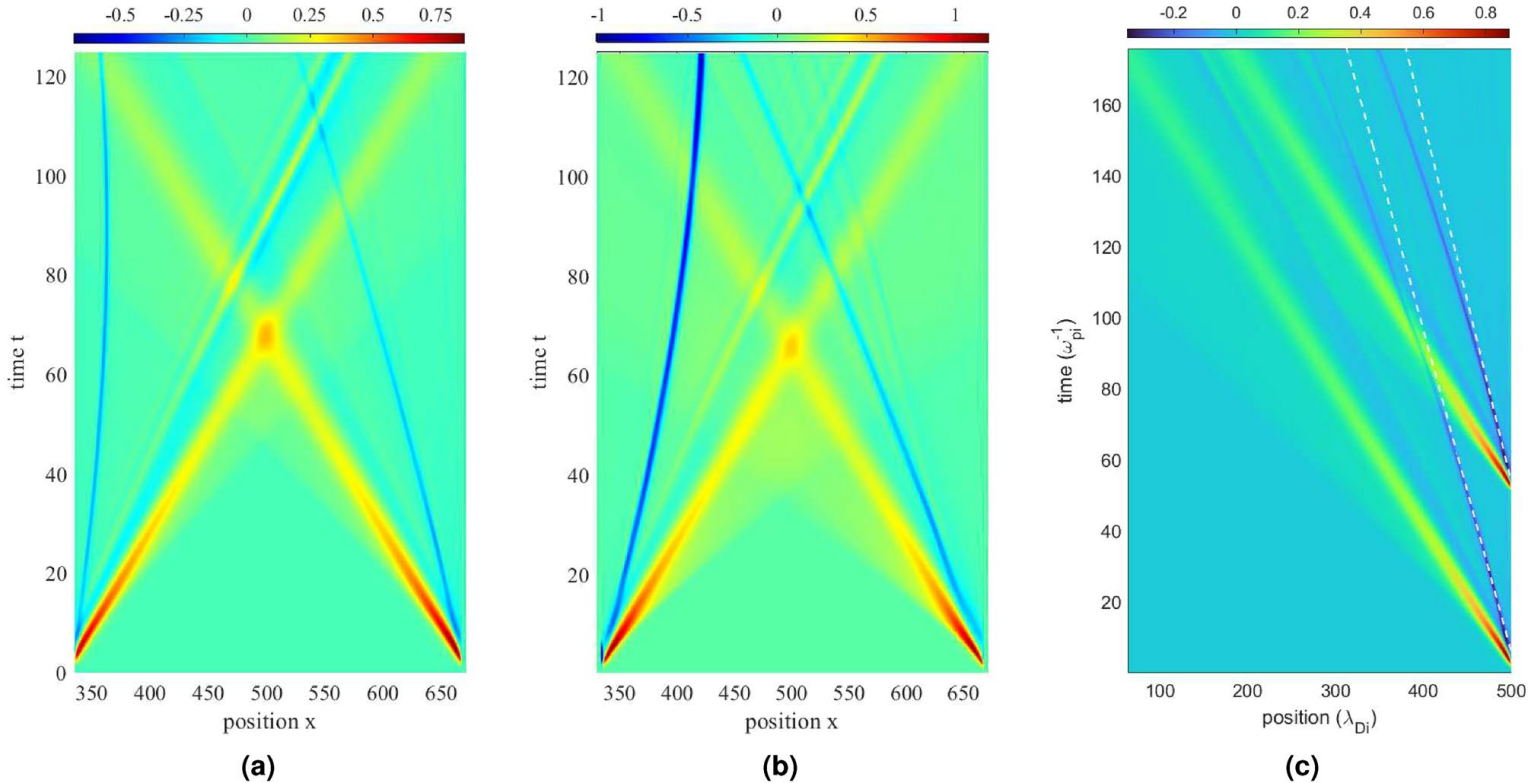
Interactions between ion phase-space hole (negative, dark blue) and ion soliton (positive, red-yellow) waves, both (**a**, **b**) representing similar interactions with different amplitudes. It can be seen that while the soliton propagates without getting affected by the hole, the ion hole gets de-accelerated or pushed backwards on the left, while the hole on the right gets pulled forward (accelerates). (**c**) Acceleration of ion phase-space holes (negative potential) due to their interactions with ion solitons (positive potential). White, dashed lines represent their initial velocity paths.

We next present head-on collisions of ion solitons and ion phase-space holes of comparable amplitudes. Using large amplitude perturbations, we simulate the propagation of ion holes under the influence of incoming solitons and observe their trajectories. It is found that ion holes tend to de-accelerate or accelerate under the influence of incoming ion KdV solitons. This interesting behaviour is shown in Fig. 6a,b. Specifically, it can be seen that when an ion phase-space hole is approached by an ion KdV soliton from the right, it experiences a de-acceleration, or gets ‘pushed back’, exhibiting a reflection-like behaviour (as seen from the backward-curve of the ion hole trajectory). On the other hand, an ion phase-space hole, when approached by an ion soliton from the left, experiences a pull and gets accelerated towards it. A similar behaviour can be observed for over-taking interactions between the two waves, in which the holes now get accelerated due to the solitons, which propagate away from the holes towards their left, as shown in Fig. 6c. For the same, perturbation pulses are introduced into the system with different amplitudes, same location and after a time interval.

In the interactions presented above, the ion hole, in the presence of an approaching or separating ion KdV soliton of sufficiently large amplitude, gets attracted or repelled, depending on the direction of motion the hole. This interaction presents an interesting case of hole acceleration and reflection, which can be explained by a mechanism similar to the the existing theories of phase-space hole self-acceleration^14,34–36^. We discuss this mechanism in the next section. We do not observe any phase-shift during the collisions of an ion hole with a KdV soliton during our study.

## Discussion

**Figure 7:**
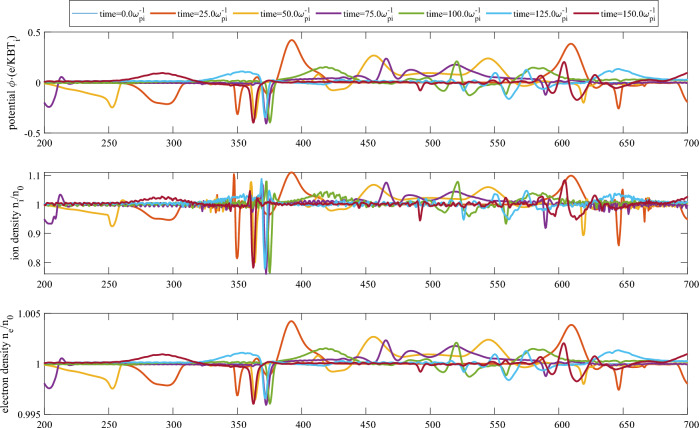
Time-evolution of (top) spatial potential profile, (middle) ion density and (bottom) electron density during head-on collisions of ion holes and ion solitons.

The acceleration of the ion hole which is evidently shown in the previous section is somewhat similar in mechanism to the hole self-acceleration phenomena, which is well-known to occur due to particle reflections and negative-mass interactions of the phase-space hole with the free and background particle distribution^34–36^. This dual nature of the hole acceleration, during its close proximity with nearly symmetric travelling electrostatic waves can be associated with three visible changes in this region, as shown in Fig. 7:

I. A net reduction in the ion density during the interaction,
II. A consequential reduction in the electron density during the interaction,
III. A shift in the phase-space density $f(x,v_x)$ along the $(-v_x)$ axis of the ion phase-space.

Another condition which we find necessary for such an interaction to occur between the ion phase-space hole and the ion soliton is that the soliton wave amplitude must be sufficiently large. This interaction can be qualitatively explained by the particle density shifts and the resultant shift of the phase-space hole depression along the velocity axis. We first discuss this mechanism for the head-on collision and resultant ion hole accelerations.

### Interaction mechanism of head-on collisions of ion hole and ion soliton

**Figure 8:**
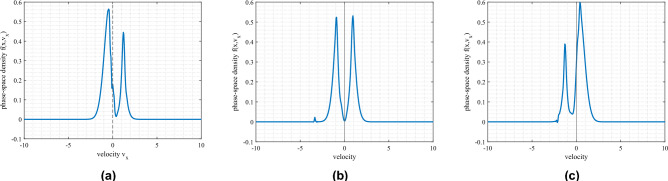
Shift of the ion phase-space density during ion hole reflection against an ion soliton. (**a**) just before reflection (time $=37.5\omega _{pi}^{-1}$), (**b**) during reflection (time $=100.0.0\omega _{pi}^{-1}$) and (**c**) after reflection (time $=168.75\omega _{pi}^{-1}$). Free ion peak shift from $v_x=-0.5$ to $v_x=0.5$ (in units of ion thermal speed), at the same position.

The ion soliton, which can be regarded as a particle accelerating field, interacts with both the streaming and the trapped ions on either side of the $v_x=0$ axis of the phase-space plane, and causes an acceleration of the free ions along its own direction. This shifts the average velocity of the ions towards the wave direction, causing the peak of the free particle distribution to shift toward the $-v_x$ axis. This shift is shown in Fig. 8.

**Figure 9:**
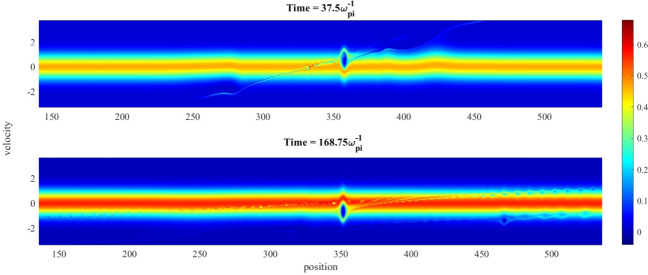
Shift of an ion hole (above) before and (below) after getting reflected against an incoming ion soliton.

This shift of the free ion distribution peak is characterised by a velocity peak-to-peak jump of $|\Delta v_x|\ge 2\sqrt{\phi _0^H(x)}$, in normalised terms, where $\phi _0^H$ is the absolute value of the hole amplitude. The soliton causes a further acceleration of the trapped particles along the wave direction. This causes a net decline in the trapped particle density in the $v_x<0$ region due to particles which escape this trapped region form the hole boundary, along-with a balancing growth of particle density in the $v_x>0$ region of the probability space due to the de-acceleration experienced by these ions. Consequently, the ion distribution in the trapped region also shifts its minima across the $v_x=0$ axis of the phase-space. This “jump” of particles from one side of the $v_x=0$ line to the other is facilitated by the electric field of the travelling soliton, and the shifting time can be calculated approximately by –

8 $$\begin{aligned} |\Delta v_x| \simeq 2\sqrt{\phi _0^H(x)} = \int _0^{\delta t}E_s(x,t)dt \simeq \frac{\phi _0^S}{2\Delta _s}\delta t , \end{aligned}$$

where $\delta t$ is the approximate shifting time of the ion hole, $E_s(x,t)$ is the soliton electric field, $\phi _0^S$ the soliton potential and $\Delta _s$ the soliton half-width. For the shown hole shift, in Fig. 8, in normalised terms, the free ion distribution function peak shift (along velocity axis) is $|\Delta v_x|=0.7v_{Ti}$, soliton potential is $0.15K_BT_i/e$, soliton half-width is $14\lambda _{Di}$ and the shift-time is calculated to be $\simeq 130.33\omega _{pi}^{-1}$. Due to this shift, the ion hole shifts along the $- v_x$ direction in the phase-space (see Fig. 9).

This causes the ion hole with an initial positive velocity to reflect and move opposite direction. A similar mechanism exists for the case of a negative velocity ion hole colliding head-on against an ion soliton. In this case, the ion acceleration caused by the ion soliton (which is now moving with a positive velocity along $+x$ axis) causes an acceleration of the free ions along the $+v_x$ axis. This is also accompanied by the acceleration of some trapped particles along the same direction, thus forcing the ion phase-space hole depression in its velocity distribution further down the $-v_x$ axis, in a region of reduced particle phase-space density and thus accelerates into a larger speed. This interaction mechanism causes acceleration of ion holes in overtaking collisions with ion KdV holes of opposite potentials, as-well-as de-accelerations and reflections during head-on collisions. This acceleration is independent of the interactions between two ion holes, as is clear from Fig. 6c.

### Interaction mechanism of overtaking collisions between ion holes and ion solitons

**Figure 10:**
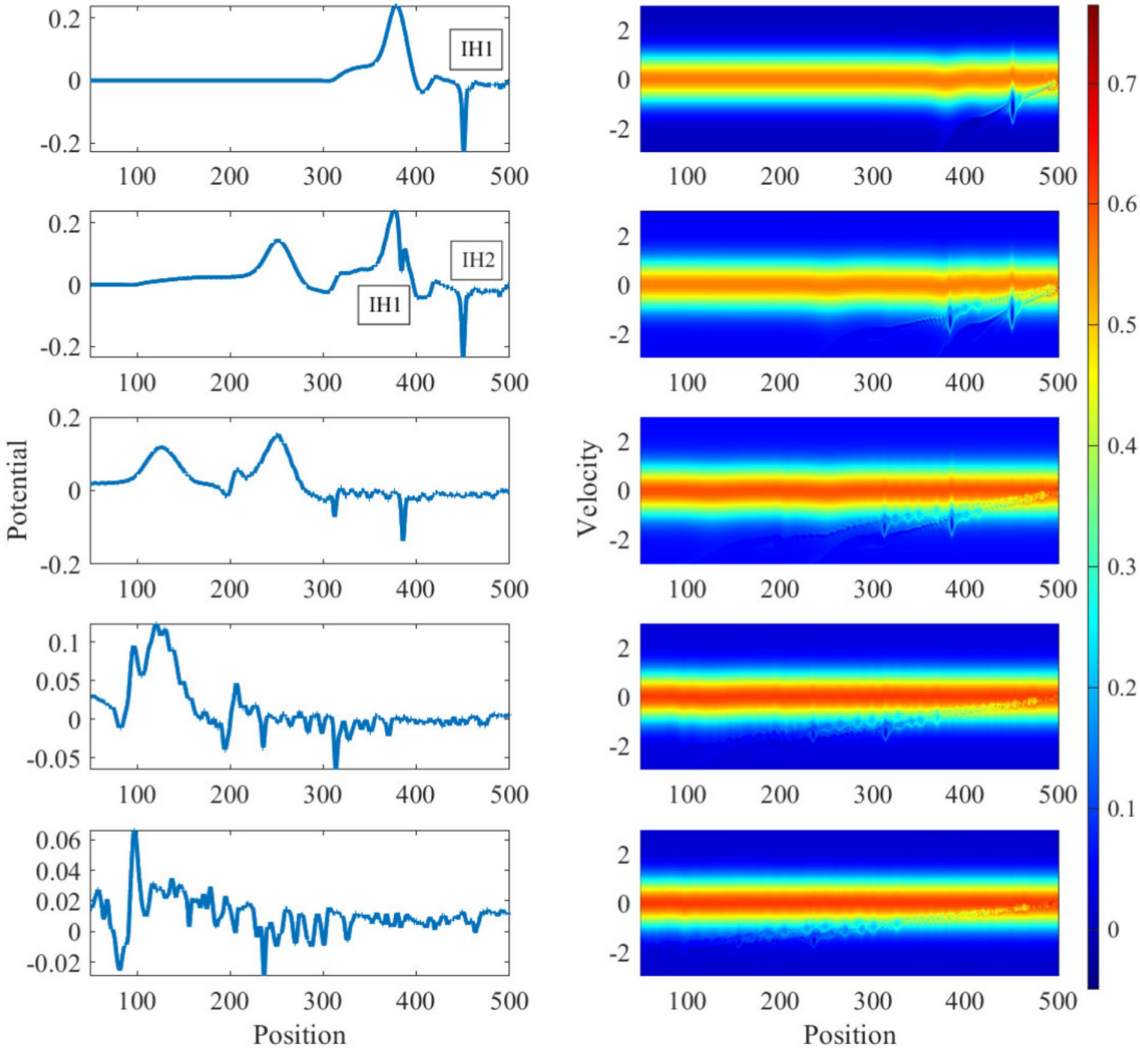
Evolution of (left) spatial potential field and (right) ion phase-space during overtaking interactions of the ion solitons and ion holes. Portraits at time intervals of $50\omega _{pi}^{-1}$ (from top to bottom).Top-most plot at time $t=50.0125\omega _{pi}^{-1}$.

For the case of over-taking interactions between the ion phase-space hole and the ion soliton, as shown in Figs. 6c and 10, the acceleration mechanisms are slightly different in nature. It can be seen that:

I. the ion hole generated initially (IH1), travels with a constant speed till it gains close proximity with the later-formed ion soliton, which is of a fairly large amplitude during its collision with the ion hole.
II. It is also evident that this ion hole experiences more acceleration than the later-formed ion hole (IH2), which can be seen from the shifts in their trajectories from their initial paths.
III. Also, both IH1 and IH2 experience significantly less accelerations during this interaction, when compared with the head-on case.

IH1 experiences a downward shift in the velocity space due to the action of the (overtaking) accelerating ion soliton, which initially produces a net flux of ions along the negative $v_x$ direction, and then accelerates them out of the trapped region, creating a particle depression in the velocity-space below the initial distribution. This causes the ion hole to momentarily jump upwards towards the $v_x=0$ line and later fall downwards along the $(-v_x)$ direction, further below its original position. The secondary ion hole IH2 follows the same mechanism produced by the initially produced soliton, which is, however, much damped and far, and hence, produces a significantly less accelerating effect on it.

### No interactions between lagging hole and leading soliton pair formed together

In the above interactions, the ion holes do not interact with the solitons produced from the same perturbation. This is due to the nature of the ion soliton formed in pair with the ion hole. The hole-soliton pair are formed in such a way that:

I. The ion hole and the ion soliton both move in the same direction, and
II. The ion soliton is much faster than the ion hole, and therefore always remains ahead.

This implies that the ion beam accelerated by the ion soliton travels in the direction along the soliton, and does not interact with the ion hole propagating much behind it. Therefore, the ion hole moves with a constant speed along the plasma, unaffected by the leading ion soliton.

### Minimum conditions for hole acceleration

According to the interaction mechanisms presented above, it can be easily deduced that for the hole acceleration (or reflection), a sufficiently large ion soliton amplitude must be present. This can be shown from the hole-soliton shift time $\delta t^I$ calculated in Eq. (8):

9 $$\begin{aligned} \delta t^I \propto \frac{\Delta _s\sqrt{\phi _0^H}}{\phi _0^S} \propto \frac{\Delta _s\cdot |\Delta v_x|}{\phi _0^S}. \end{aligned}$$

It is clear that this shift time will be small for sufficiently large soliton amplitude $\phi _0^S$, and increases considerably for small amplitude ion solitons, also due to the inverse relationship between soliton amplitude and width $(\Delta _s)$. This shifting time must be sufficiently small to produce a significant hole shift along the velocity space, during the actual hole-soliton interaction time $\delta t_I$, which is approximately equal to $\Delta _s/(M_s-M_H)$. Furthermore, a significant shift in the hole distribution along velocity space would require a sufficiently strong accelerating field, which is directly dependent on the soliton amplitude. Hence, the interaction between ion hole and ion soliton must require the two waves to have sufficiently strong potential amplitudes and similar speeds.

These interactions indicate strong interactions between the trapped and free ions, which interact due to the existing fields. In classically-analogous terms, the free ions, subject to the direction of their acceleration by the travelling KdV soliton, ‘kick-out’ the trapped particles from the hole region. This causes a change in the phase-space particle densities along the velocity axis, therefore causing a ‘shift’. This shift then directly corresponds to the hole acceleration (or de-acceleration), which is observed in the presented simulation study.

## Data Availability

All data generated during the study conducted in this work are available with the corresponding author (Allen Lobo) upon reasonable request.
